# Data on the effects of a vertical agrivoltaic system on crop yield and nutrient content of barley (*Hordeum vulgare L.*) in Sweden

**DOI:** 10.1016/j.dib.2024.110990

**Published:** 2024-10-09

**Authors:** S. Ma Lu, S. Zainali, T.E.K. Zidane, T. Hörndahl, S. Tekie, A. Khosravi, M. Guezgouz, B. Stridh, A. Avelin, P.E. Campana

**Affiliations:** a Mälardalen University, Department of Sustainable Energy Systems, Västerås, Sweden; b Swedish University of Agricultural Sciences, Department of Biosystems and Technology, Alnarp, Sweden

**Keywords:** Vertical agrivoltaic, Barley analysis, Dual land-use, Dataset

## Abstract

Agrivoltaic systems emerge as a promising solution to the ongoing conflict between allocating agricultural land for food production and establishing solar parks. This field experiment, conducted during the spring and summer seasons of 2023, aims to showcase barley production in a vertical agrivoltaic system compared to open-field reference conditions at Kärrbo Prästgård, near Västerås, Sweden. The dataset presented in this article encompasses both barley kernel and straw yields, kernel crude protein levels, starch content in kernels and thousand kernel weight. All collected data underwent analysis of variance (ANOVA) with Tukey pairwise comparison when possible, using dedicated software RStudio 4.3.2. This dataset article illustrates the effects of the vertical agrivoltaic design system on barley productivity. Interested researchers can benefit from this data to better comprehend barley yield under this specific agrivoltaic design and conduct further analyses and comparisons with yields from different locations or design configurations. The experimental data holds the potential to foster collaborations and advance research in agrivoltaic systems, providing a valuable resource for anyone interested in the subject. It was observed that the mean barley yield in all the different areas of the vertical agrivoltaic system were higher than the one in the control area. Additionally, weather and solar irradiance data collected during the growing season are provided in the repository for further usage.

Specifications Table

**Table:** 

Subject	Agronomy and Crop Science and, Renewable Energy, Sustainability and the Environment.
Specific subject area	Agriculture and Solar Photovoltaics
Type of data	Tables and Figures. Analyzed mean and raw data.
Data collection	Data of barley related to yield kernels and straws (kg DM/ha), nitrogen content in kernels (%), crude protein in kernels (%), kernels yield (kg DM/ha), straws yield (kg DM/ha), starch content in kernels (%), and thousand kernel weight (%) were obtained during the harvest on September 7, 2023, at Kärrbo Prästgård, Sweden. Data were collected in five groups, each consisting of two subgroups. In each subgroup, squared samples (each 0.25 m^2^) were collected with five replications, totaling fifty samples. These samples were statistically analyzed as described in this article.
Data source location	Mälardalen University, Sweden, is the owner of the data presented in this article. The experimental site is located at Kärrbo Prästgård with 59.55° N latitude, 16.76° E longitude, and altitude of 21 meters above sea level.
Data accessibility	Repository name: Zenodo Data identification number: 10.5281/zenodo.12655139 Direct URL to data: https://zenodo.org/doi/10.5281/zenodo.12655139 Instructions for accessing these data: None
Related research article	None

## 1 Value of the Data

• This dataset provides insights on the effect of a vertically mounted agrivoltaic (APV) system on barley yield, crude protein, and starch content in Sweden, offering comparisons with barley grown in open-field (reference) conditions as well as in a conventional ground-mounted fixed-tilt photovoltaic (PV) system. This data enriches the research community's understanding of barley performance and its response under this specific system design in Nordic countries.
• The methodology outlined here for crop experiments, harvesting, and analysis can be replicated for other sites. It not only serves as a guide but also inspires and encourages further research, exploring new system configurations and crop varieties for comprehensive comparisons.
• The dataset offers valuable insights into the viability of vertically mounted APVs in Sweden, considering the production level of barley as crop. It can serve to enlighten policymakers about the potential and feasibility of dual land use, enabling them to implement appropriate measures to encourage adoption (e.g., subsidies or legislation regarding crop yield reduction limits). Furthermore, it can support PV developers in the permitting process by presenting concrete evidence of these systems’ crop performance.
• Despite the location-, system design-, and crop-dependent nature of APVs, integrated models consider all these characteristics and their interactions to predict performance accurately. This dataset becomes a valuable resource for researchers aiming to advance and validate crop models tailored for APVs. Additionally, this work promotes the sharing of field experiment data to foster collaborative research efforts. In doing so, it significantly contributes to the overall research advancement of APV technology.

## 2 Background

The European Union targets net-zero greenhouse gas emissions by 2050 [1], with a substantial focus on widespread solar photovoltaic (PV) deployment. Ground-mounted PV systems compete for land with agriculture, but APV systems enable dual land use for both PV systems and agriculture. Over the past decade, research has intensified to implement APV systems. However, studies reveal that APV performance depends on climate and system design [2]. Laub et al.’s [3] meta-analysis highlights yield response curves for various crops under different light levels but notes limitations, including the lack of interaction data with factors like water availability, varying APV system designs (e.g., vertically mounted systems) and the independent nature of experiments (testing only one crop type in a given location).

This dataset examines barley performance within a vertically mounted APV system in Sweden. APV systems can stimulate rural economic development [4] by providing farmers dual revenue streams from crops and electricity [5]. Successful adoption relies on tangible, positive results from field tests, crucial for farmers, policymakers, investors, and legislators. These tests must occur seasonally due to APV systems’ dependence on crop growth and system size, focusing on specific crops each season. Integrated modeling tools are essential for predicting site potential before installation [5] and must be validated with real experimental data to ensure reliability.

## 3 Data Description

**Fig. 1:**
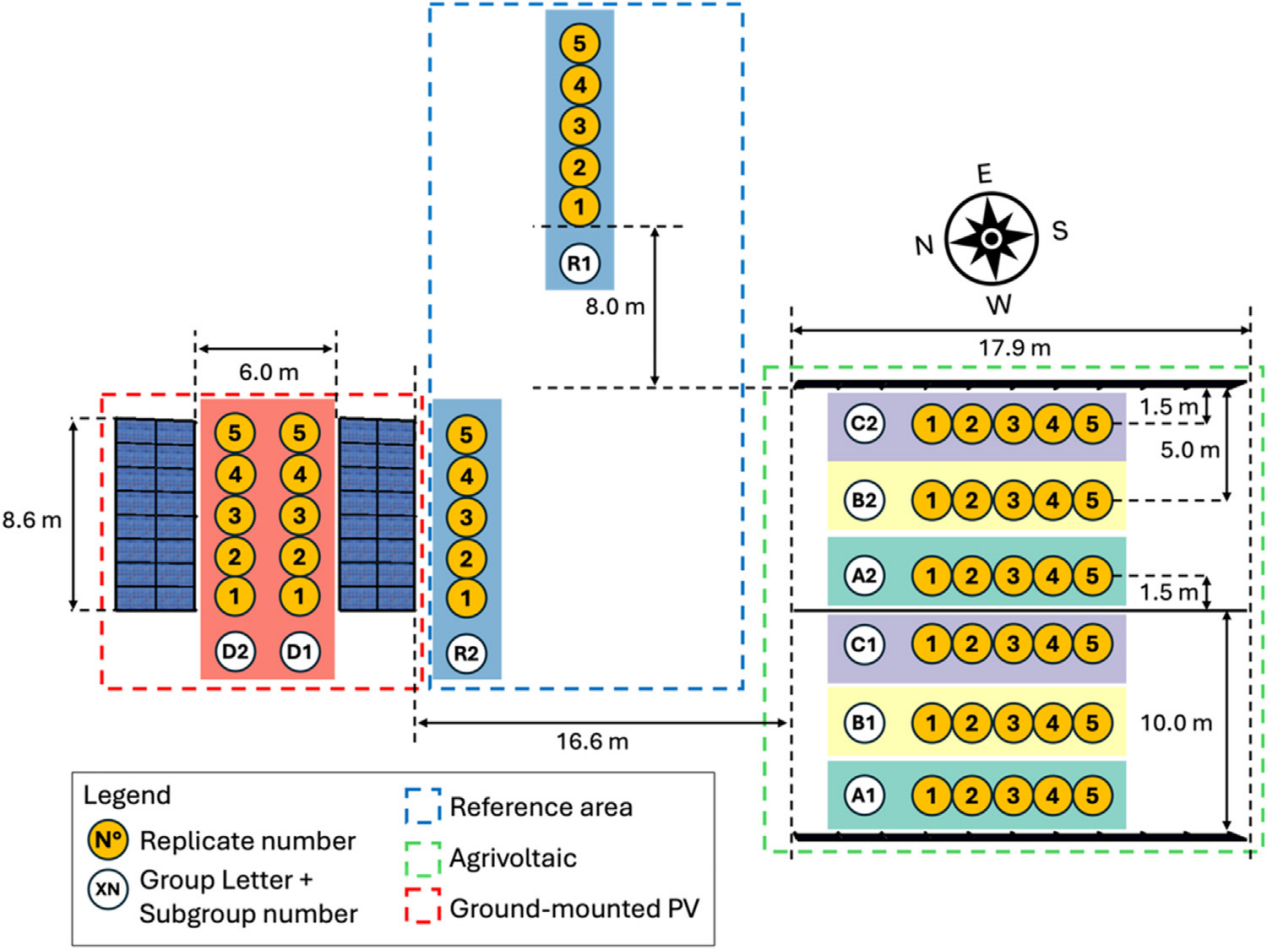
Crop experiment layout (top view) of the vertical agrivoltaic system and conventional ground-mounted system in Kärrbo Prästgård, Sweden. The illustration is not to scale.

This dataset originates from a field experiment conducted throughout the primary cropping season, spanning May to September 2023, at Kärrbo Prästgård (59.55° N, 16.76° E), near Västerås, Sweden (Fig. 1). The location is home to Sweden's first APV system, established in 2021, which underwent two initial years of field experiments involving ley grass [5]. As of 2023, the APV experimental site adheres to a conventional Swedish crop rotation, with the late spring and summer seasons of 2023 dedicated to barley cultivation.

The dataset provides measurements of the harvest as well as statistical crop analysis of the parameters collected: yield, nitrogen content, crude protein levels, kernel yield, straw yield, starch content in kernels, and thousand kernel weight (TKW). The statistical analysis, described in further detail in the next section, encompassed a systematic approach, employing five distinct groups (Fig. 1), each serving a specific purpose:

1. Group A: Edge plots on the west side of the APV system's rows.
2. Group B: Middle plots within the APV system's rows.
3. Group C: Edge plots on the east side of the APV system's rows.
4. Group D: Plots within the conventional ground-mounted PV.
5. Group R: Control plot (Reference area / open-field).

**Fig. 2:**
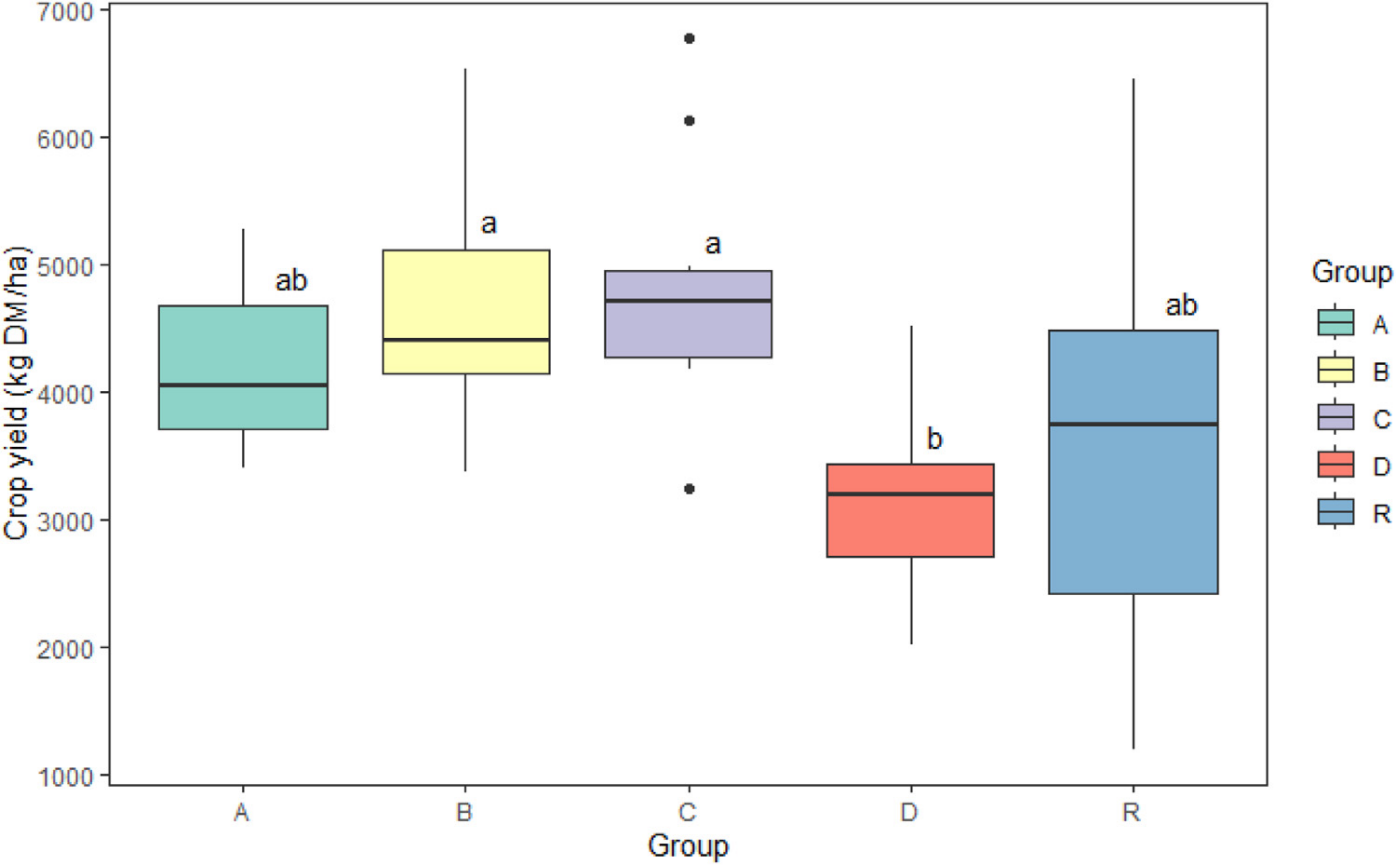
Statistical analysis for yield of kernel and straw (kg DM/ha) for Kärrbo Prästgård 2023 using balanced one-way ANOVA with Tukey pairwise comparison with a 95% confidence. Different letters above the bars indicate significant differences between groups. Groups sharing the same letter are not significantly different from each other at the 95% confidence level.

The analysis of yield of kernel and straw (kg DM/ha) revealed significant differences among the groups (Fig. 2, Table 1). The balanced one-way ANOVA with Tukey pairwise comparison (95% confidence) indicated that Group B and Group C exhibited higher mean yield, while Group R had lower mean yield and a large spread. Group D had the lowest.

**Table 1:** Statistical analysis for yield of kernel and straw (kg DM/ha) for Kärrbo Prästgård 2023 using balanced one-way ANOVA with Tukey pairwise comparison with a 95% confidence. Different superscript letters indicate significant differences between groups. Groups sharing the same letter are not significantly different from each other at the 95% confidence level.

Block	Mean	Median	SD	Lower Quantile	Upper quantile	Min	Max
A	4191^ab^	4040	672	3697	4667	3395	5266
B	4612^a^	4403	955	4136	5111	3372	6526
C	4826^a^	4696	1000	4272	4952	3230	6767
D	3122^b^	3188	724	2704	3426	2009	4506
R	3499^ab^	3739	1640	2408	4476	1193	6439

The Welch-ANOVA with Games-Howell pairwise comparison (95% confidence) for yield kernel (kg DM/ha) indicated significant differences among the groups, as outlined in Table 2. Specifically, Group C demonstrated the highest mean yield kernel, while Group D recorded the lowest.

**Table 2:** Statistical analysis for yield kernel (kg DM/ha) for Kärrbo Prästgård 2023 using Welch-ANOVA with Games-Howell pairwise comparison with a 95% confidence. Different superscript letters indicate significant differences between groups. Groups sharing the same letter are not significantly different from each other at the 95% confidence level.

Block	Mean	Median	SD	Lower Quantile	Upper quantile	Min	Max
A	2083^a^	2009	351	1868	2322	1487	2571
B	2335^a^	2164	544	2018	2660	1670	3394
C	2379^a^	2382	455	2084	2423	1704	3157
D	1456^b^	1521	369	1254	1664	880	2053
R	1784^ab^	1940	841	1080	2281	689	3331

For yield straw (kg DM/ha), the balanced one-way ANOVA with Tukey pairwise comparison (95% confidence) highlighted significant variations among the groups (Table 3). Group C exhibited the highest mean yield straw, while Group D displayed the lowest.

**Table 3:** Statistical analysis for yield straw (kg DM/ha) for Kärrbo Prästgård 2023 using balanced one-way ANOVA with Tukey pairwise comparison with a 95% confidence. Different superscript letters indicate significant differences between groups. Groups sharing the same letter are not significantly different from each other at the 95% confidence level.

Block	Mean	Median	SD	Lower Quantile	Upper quantile	Min	Max
A	2109^ab^	2074	423	1920	2242	1386	2824
B	2277^ab^	2195	452	2032	2492	1640	3132
C	2447^a^	2444	566	2114	2547	1526	3609
D	1665^b^	1630	378	1422	1801	1129	2453
R	1715^b^	1799	819	1327	2232	485	3108

The analysis of crude protein in kernel (%) using the Kruskal-Wallis test with Wilcoxon pairwise comparison (95% confidence) revealed significant variations across the groups, as summarized in Table 4. Notably, Group R exhibited the highest mean crude protein content, while Group D displayed the lowest.

**Table 4:** Statistical analysis for crude protein in kernel (%) for Kärrbo Prästgård 2023 using Kruskal-Wallis test with Wilcoxon pairwise comparison with a 95% confidence. Different superscript letters indicate significant differences between groups. Groups sharing the same letter are not significantly different from each other at the 95% confidence level.

Block	Mean	Median	SD	Lower Quantile	Upper quantile	Min	Max
A	13.1^ab^	13.2	0.36	12.8	13.3	12.5	13.7
B	13.4^ab^	13.3	0.519	13.2	13.4	12.9	14.8
C	13.0^a^	12.9	0.406	12.8	13.4	12.3	13.4
D	11.8^c^	11.8	1.26	10.6	12.6	10.3	13.9
R	13.6^b^	13.6	0.497	13.2	13.8	12.8	14.4

Turning to starch in kernel (%), the Kruskal-Wallis test with Wilcoxon pairwise comparison (95% confidence) revealed substantial differences among the groups, as summarized in Table 5. Group D exhibited the highest mean starch content in kernels, in contrast to Group R, which had the lowest.

**Table 5:** Statistical analysis for starch in kernel (%) for Kärrbo Prästgård 2023 using Kruskal-Wallis test with Wilcoxon pairwise comparison with a 95% confidence. Different superscript letters indicate significant differences between groups. Groups sharing the same letter are not significantly different from each other at the 95% confidence level.

Block	Mean	Median	SD	Lower Quantile	Upper quantile	Min	Max
A	59.4^a^	59.4	0.36	59.2	59.7	58.7	59.9
B	59.1^ab^	59.1	0.59	58.7	59.5	57.9	59.9
C	58.8^b^	58.8	0.52	58.6	59	57.8	59.8
D	60.6^c^	60.8	1.28	59.7	61.7	58.5	62.2
R	58.2^d^	58.2	58.2	58	58.6	57.2	58.9

Lastly, the Kruskal-Wallis test with Wilcoxon pairwise comparison (95% confidence) for TKW indicated significant variations among the groups (Table 6). Group D demonstrated the highest mean TKW, while Group C had the lowest.

**Table 6:** Statistical analysis for TKW (%) for Kärrbo Prästgård 2023 using Kruskal-Wallis test with Wilcoxon pairwise comparison with a 95% confidence. Different superscript letters indicate significant differences between groups. Groups sharing the same letter are not significantly different from each other at the 95% confidence level.

Block	Mean	Median	SD	Lower Quantile	Upper quantile	Min	Max
A	42.1^a^	42.2	1.3	41.6	43	39.8	43.7
B	42.1^ab^	42	1.6	40.7	43.5	40.2	44.4
C	41.3^a^	41.3	2.1	39.8	42	38.1	45.4
D	45.1^c^	45.2	0.9	44.6	45.6	43.2	46.2
R	43^b^	43.8	3.08	42.9	44.5	34.7	45.3

## 4 Experimental Design, Materials and Methods

**Fig. 3:**
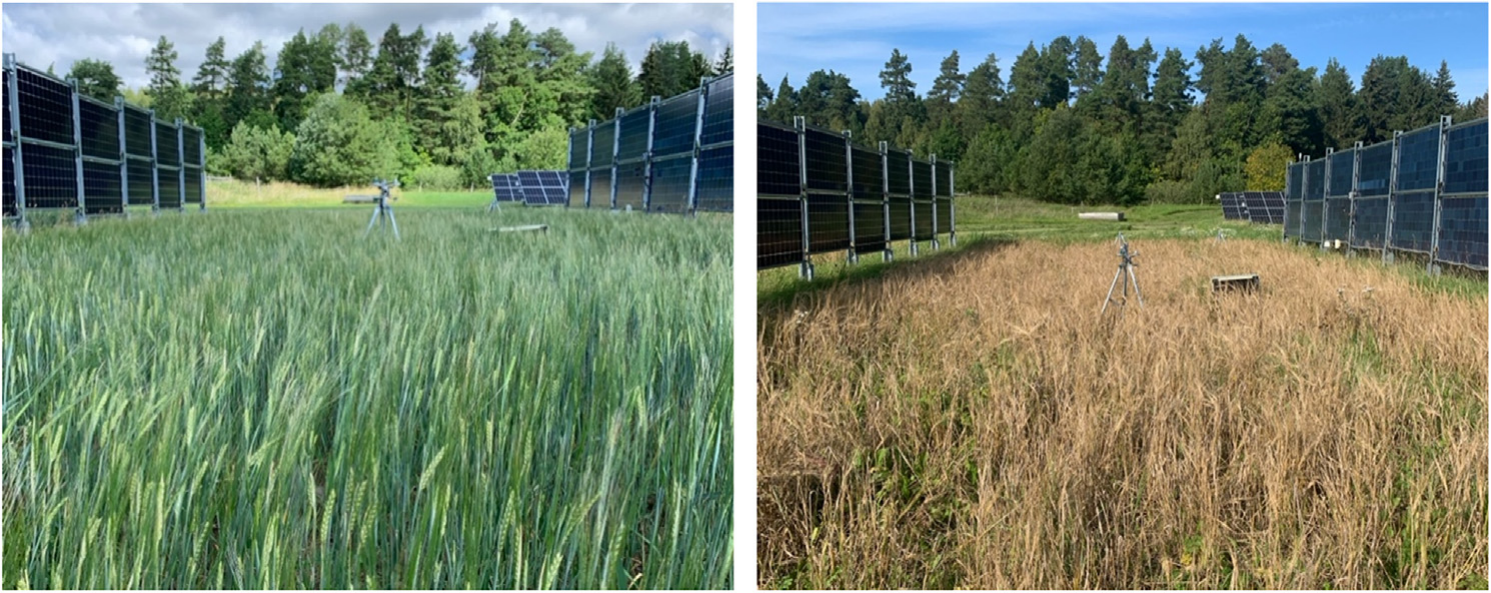
Barley growing in between the vertically mounted APV system in Kärrbo Prästgård 2023. Left: July 14. Right: September 6.

Barley (*Hordeum vulgare L*. variety Dragon [6]) was sown on the APV site on the May 12, 2023 at a rate of 220 kg/ha. Additionally, nitrogen (consisting of equal parts ammonium and nitrate nitrogen) fertilizer with a moderate sulfur content (Axan NS 27-4, YaraBela) was used at a rate of 220 kg/ha. Finally, 60 kg/ha of nitrogen organic fertilizer (Biofer, Gyllebo Gödning) was added at a depth of 4 cm. Thereafter, the field has been left to grow naturally without irrigation or any other agricultural practices (Fig. 3).

To study the effects of the vertically mounted APV system as well as the conventional ground-mounted PV system on barley, fifty samples were distributed in five groups, as shown in Fig. 1. Each group consisted of two subgroups. In each subgroup, squared samples were collected with five replications. Groups A, B and C are based on the spatial location in the crop area between two vertical rows of PV modules: west side (A), center side (B), and east side (C). This design is thought to allow for a more in-depth study of the various spatial locations where crops can grow in a vertical APV system. Group R corresponds to the reference control plot conditions. While group D represents the crops that would be growing in the space between two rows of conventional ground-mounted PV systems. For further information on the APV system size and characteristics, the reader is referred to [5].

### 4.1 Crop data collection

The samples were hand-harvested five centimeters from the ground on September 7, 2023, with each sample corresponding to a square area of 0.25 m^2^. During collection, weeds were meticulously removed from the samples, and only the kernels and straws from the barley were retained and divided for further analysis (Fig. 3).

### 4.2 Yield of kernel and straw

The fresh weight (kg/ha) of the samples (kernel and straw together) was measured immediately after cutting. Subsequently, the samples were dried at 60°C for 24 hours and weighed again (both kernel and straw together and separately) to determine the dry matter (DM) content (%). The yield of kernel and straw (kg DM/ha) was then calculated accordingly. An approximation of the same amount of water content in the kernels and straws at cut was assumed.

### 4.3 Nutrient Content Analysis

The method used to determine moisture and protein in whole barley kernels adheres to the European Standard EN 15948:2010. This method utilizes Near-Infrared Transmittance (NIT) combined with an Artificial Neural Network (ANN) prediction model and an associated database. The NIT instrument employed is a Grain Analyzer (Infratec 1242 by FOSS). The calibration model used is the one endorsed for large-scale applications by the Swedish Food Agency. Through this method, analyses of crude protein and starch content in the kernels were conducted.

### 4.4 Thousand kernel weight

TKW is measured using OPTO-AGRI (Opto Machines). This process involves placing the sample in a tray and employing image processing to count the number of kernels and determine their weight.

### 4.5 Statistical data analysis

To ensure the reliability of the data, an initial assessment involved examining residual plots and distribution plots, leading to the identification of the necessity for data transformation. Furthermore, to validate the assumptions of normality and equal variances between groups, Levene and Shapiro-Wilk tests were conducted. If these assumptions were not violated, a balanced one-way analysis of variance (ANOVA) was performed using Tukey's honest significance test with a confidence level of 95%. In instances where the assumption of equal variances was compromised, a Welch ANOVA accompanied by the Games-Howell test with a confidence level of 95% was implemented. If neither normal distribution nor equal variances were observed, a Kruskal-Wallis test was employed, followed by a Wilcoxon pairwise test with a confidence level of 95%.

In the analysis, RStudio version 4.3.2 was used as the preferred integrated development environment, known for its effectiveness in performing statistical calculations and data analyses.

## Limitations

The weather patterns during the barley growing season from May to September 2023 in Sweden were notably atypical. For instance, June experienced a dry and hot weather, prompting the implementation of fire bans. Conversely, July and August were characterized by heavy rainfall [7], recording a total of 165.0 mm and 189.3 mm of rainfall onsite, respectively. According to data from the Swedish Meteorological and Hydrological Institute (SMHI), Västerås (the closest city to the experimental facility) witnessed its wettest July since 2000, recording a total of 156.8 mm of rainfall [8]. These anomalous climatic events have the potential to influence the growing season and consequently impact the yield as well as the findings presented in this study. It is imperative to be aware of these factors when further analyzing and interpreting the results. For the convenience of the research community, the repository includes measured weather and solar irradiance parameters collected during the growing season at an hourly resolution. These parameters are: air temperature (°C), relative humidity (%), relative air pressure (hPa), wind speed (m/s), precipitation (mm/h), global horizontal irradiance (W/m^2^), diffuse horizontal irradiance (W/m^2^), and photosynthetically active radiation (µmol/m^2^/s). Additionally, readers should note another limitation of this study: the small number of samples and replicates. In the broader field of agronomy, and particularly in APV systems, the number of replicates is often constrained by the size of the system, especially in non-commercial research facilities. As a result, it is common practice to have a limited number of replicates in such studies (cf. [9]).

## Ethics Statement

The dataset collected in this study did not involve animals, humans or any data collected from social media platforms.

## CRediT authorship contribution statement

**S. Ma Lu:** Conceptualization, Methodology, Investigation, Formal analysis, Visualization, Writing – original draft, Writing – review & editing. **S. Zainali:** Conceptualization, Methodology, Investigation, Formal analysis, Visualization, Writing – original draft, Writing – review & editing. **T.E.K. Zidane:** Writing – original draft, Writing – review & editing. **T. Hörndahl:** Methodology, Formal analysis, Writing – review & editing. **S. Tekie:** Writing – review & editing. **A. Khosravi:** Writing – original draft, Writing – review & editing. **M. Guezgouz:** Writing – review & editing. **B. Stridh:** Writing – review & editing, Funding acquisition. **A. Avelin:** Writing – review & editing. **P.E. Campana:** Funding acquisition, Conceptualization, Methodology, Writing – review & editing.

## Data Availability

ZenodoData on Crop Yield, Nutrient Content of Barley (Hordeum vulgare L.) and Weather of a Vertical Agrivoltaic System in Sweden (Original data).
